# A mild case of Cockayne syndrome with a novel start-loss variant of *ERCC8*

**DOI:** 10.1038/s41439-024-00297-6

**Published:** 2024-11-07

**Authors:** Taro Matsuoka, Takeshi Yoshida, Kengo Kora, Naoko Yano, Yoshihiro Taura, Takashi Nakamura, Takenori Tozawa, Jun Mori, Tomohiro Chiyonobu

**Affiliations:** 1 Department of Pediatrics, Graduate School of Medical Science, Kyoto Prefectural University of Medicine, Kyoto, Japan; 2 Department of Pediatrics, Kyoto University Graduate School of Medicine, Kyoto, Japan; 3 Department of Otolaryngology-Head and Neck Surgery, Kyoto Prefectural University of Medicine, Kyoto, Japan; 4 Division of Pediatric Endocrinology, Metabolism and Nephrology, Children’s Medical Center, Osaka City General Hospital, Osaka, Japan; 5 Department of Molecular Diagnostics and Therapeutics, Graduate School of Medical Science, Kyoto Prefectural University of Medicine, Kyoto, Japan

**Keywords:** Neurodegeneration, Genetic counselling

## Abstract

Cockayne syndrome (CS) is a progressive multisystem disorder characterized by growth failure, microcephaly, developmental delay, and photosensitivity. The characteristic symptoms appear during early childhood in most patients with CS. Herein, we report a mild case of CS with a novel start-loss variant in *ERCC8* that did not show the characteristic symptoms of CS during early childhood and exhibited sudden growth failure after the age of 10 years.

Cockayne syndrome (CS) (OMIM #216400 and #133540) is a progressive multisystem disorder characterized by growth failure, microcephaly, developmental delay, and photosensitivity. CS is typically caused by biallelic loss-of-function variants of *ERCC8* (CS type A) or *ERCC6* (CS type B). Although there are no specific disease-modifying therapies for CS, early diagnosis and management of complications are desirable because CS exhibits various systemic symptoms. Most patients show significant growth failure by the age of 2 years. Additionally, it is accompanied by characteristics, such as microcephaly, developmental delay, cutaneous photosensitivity, cataracts, sensorineural hearing loss, enamel hypoplasia, and enophthalmia, making it possible to diagnose the disease^1,2^. However, the clinical diagnosis is often challenging in patients with atypical or mild phenotypes; therefore, comprehensive genetic analysis is needed for diagnosis^3^. Herein, we report a mild case of CS that presented with sudden growth failure after 10 years of age. In this patient, whole-exome sequencing (WES) identified a novel start-loss variant combined with a known complex rearrangement involving exon 4 of *ERCC8*.

The patient was a 12-year-old female. She was born at 38 weeks of gestation and her height, weight, and head circumference were 45.5 cm (−1.2 standard deviation [SD]), 2774 g (0.2 SD), and 31.5 cm (−1.1 SD), respectively, at birth. The patient had no family history of neuromuscular disease or developmental delay. She had a mild developmental delay at 3 years of age and then was diagnosed with autism spectrum disorder and mild intellectual disability at 4 years of age. At 12-year-old, she was first noted to have a short stature at a school checkup and tremors, which led to her referral to our hospital. At the initial examination, she was 135 cm in height (−2.5 SD) and 35 kg in weight (−1.0 SD), and her growth failure became apparent at around 10 years of age (Fig. 1A–C). She also had mild ataxia and kinetic tremors. She had mild enophthalmia but did not have enamel hypoplasia, areflexia, or spasticity. Computed tomography (CT) of the head revealed bilateral striatal calcifications and magnetic resonance imaging (MRI) of the head revealed cerebellar atrophy (Fig. 1D and E). Despite the absence of growth spurt and pubertal signs, the radial epiphyseal line was closed on carpal radiography. Blood tests revealed normal levels of insulin-like growth factor 1, intact parathyroid hormone, and thyroid hormone (thyroid-stimulating hormone, free triiodothyronine, and free thyroxine). Gonadal hormone (luteinizing hormone, follicle-stimulating hormone, and estradiol) levels were equivalent to those of adult females.

**Fig. 1:**
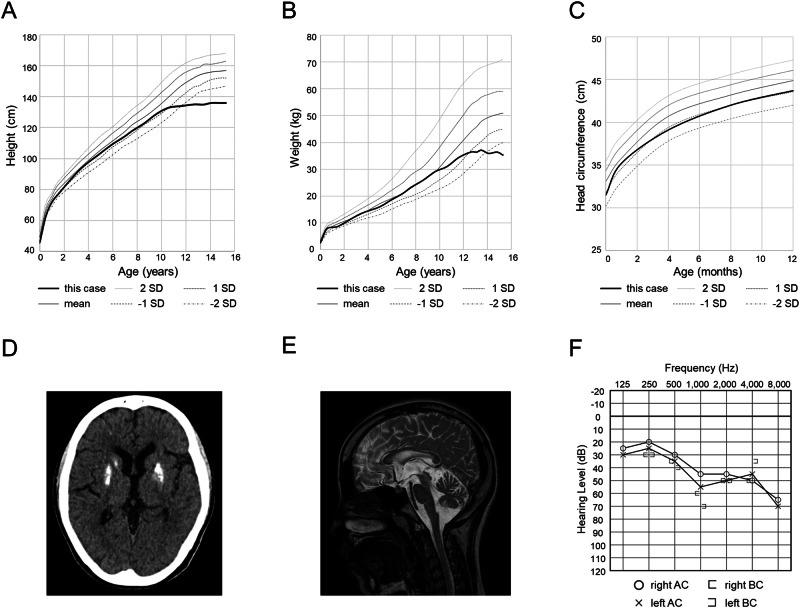
Growth charts, head image findings, and audiogram of this patient. **A**–**C** Height, weight, and head circumference of this patient compared to Japanese girls with mean and SD values^20^. **D** Head CT image showed calcification of the striatum. **E** Head MRI (T2 weighted) showed cerebellar atrophy. **F** The audiogram showed moderate bilateral sensorineural hearing loss. BC bone conduction, AC air conduction.

Trio-based WES was performed using the xGen® Exome Research Panel v2 (IDT, Iowa, USA) after written informed consent was obtained from the parents in accordance with the Kyoto University Review Committee and Ethics Committee. The imported library was sequenced using DNBSEQ-G400 (MGI Tech, Shenzhen, China). WES identified a novel heterozygous start-loss variant [NM_000082: c.1A>T, p.M1?] in *ERCC8*. The same variant was identified in the patient’s mother. Furthermore, PCR with published primers that detect a rearrangement in exon 4 of *ERCC8*, which is frequently found in Japanese patients with CS, revealed that the patient and the father have the rearrangement^4,5^. The maternal-derived variant was absent in the gnomAD, HGVD, and 8.3KJPN. This variant was predicted to be deleterious by CADD (score, 18.4; https://cadd.gs.washington.edu/), deleterious by SIFT (score, 0; https://sift.bii.a-star.edu.sg), disease-causing by Mutation Taster (probability, 0.99; http://www.mutationtaster.org), and pathogenic by PoStaL, a machine learning-based prediction tool for the pathogenicity of start-loss variants (score, 0.689; https://github.com/a-tkt/PoStaL). The pathogenicity of this variant was evaluated according to the 2015 American Society for Medical Genetics and Genomics (ACMG) guidelines and was classified as pathogenic (PVS1, PM2, PM3, PP3, and PP4)^6^. She was diagnosed with CS caused by compound heterozygous *ERCC8* variants. After diagnosis, an examination to check for complications revealed sensorineural hearing loss that the family was unaware of and started using bilateral hearing aids (Fig. 1F). A detailed interview revealed that she had mild photosensitivity and received proper guidance throughout her life. Visual impairment and renal dysfunction, which can be complicated by Cockayne syndrome, have not been seen, and the patient continues to be monitored for these findings. Although vision issues and renal dysfunction, which can be complicated in patients with CS, have not been observed, the patient is still under follow-up.

CS is classified as CS type 1 (CS1), in which symptoms such as growth failure and developmental delay appear within the first 2 years of birth; CS type 2 (CS2), a more severe form in which symptoms appear from birth; and CS type 3 (CS3), a mild or delayed form in which the first symptoms appear after 3−4 years of birth^2^. The overall incidence of CS is estimated to be 2.7/1 million people in Japan and Western Europe, of which CS3 is very rare (6.4–12.5%)^5,7,8^. The patient was classified as CS3 because the characteristic CS symptoms did not become apparent until the patient was in her teens. Our patient followed the same course as other patients with CS3. Her severity score for CS was 15 at 0-year-old, 14 at 3-year-old, 12 at 12-year-old, and 10 at 14-year-old. The severity score for CS encompasses five key items: weight and height, head circumference, neurosensorial symptoms, autonomy and motor development, and communication skills. The severity score for CS is evaluated on a 4-point scale from 0 to 3 for each item, with a total possible score of 15 points. A lower score indicates a more severe condition^9^.

Growth failure is one of the most characteristic symptoms seen in most patients with CS. CS1 shows microcephaly at 2-month-old and growth failure from 5 to 22-month-old; CS2 shows microcephaly at birth and growth failure from birth to 3-month-old^1^. Although there are few detailed reports on the clinical manifestations of CS3, some patients show growth failure before 2-year-old, while others show no growth failure at all^8,10,11^. However, there have been no cases of sudden growth failure after 10-year-old, as observed in the present case.

Although it has been assumed that there is no clear correlation between phenotype and genotype in CS, missense variants in *ERCC8* have recently been reported to result in milder phenotypes than protein-truncated variants^2,8^. In Japan and other East Asian regions, patients with CS frequently exhibit exon 4 rearrangement variants in *ERCC8*. This variant was considered the founder variant. Many patients who are homozygous for this founder variant present with typical CS and severe intellectual disability. Additionally, patients with CS with compound heterozygosity involving the founder variant and other pathogenic variants have been reported in these regions (Table 1). Due to the small number of cases, it was difficult to identify a clear relationship between the types of variants and their phenotypes. However, in this case, some of the patients had mild intellectual disability.

**Table 1:** Clinical features of CS patients with compound heterozygosity involving the founder variant and other pathogenic variants in *ERCC8*.

Country, Reference	Patient ID	Pathogenic variant (variant on an allele different from the allele with founder variant in *ERCC8*)	Protein alteration	Age at onset	Growth failure	Intellectual disability (severe/mild)	Microcephaly	Clinical classification
China, Hua et al.^17^	II:6	c.618-2A>G splicing variant (leads to an in-frame deletion of 9 bp in exon 8)	Loss of three amino acids	0 m	+	S	+	I
	II:10			?	+	−	+	I
Singapore, Ting et al.^18^	–	deletion of 277 kbp at 5q12.1 (The deletion includes the whole *ERCC8* and the first two exons of the *NDUFAF2*)	p.?	2 y	+	S	+	I
China, Wang et al.^19^	CS 01	c.394_398delTTACA	p.L132NfsX6	12 m	+	S	+	?
	CA 06	c.299insA	p.Y100X	8 m	+	S	+	?
	CA 21	c.843+2T>C	p.?	6 m	+	M	+	?
	CA 11	c.2T>A	p.M1?	?	?	?	?	?
Japan, Sugita et al.^16^ Calmels et al.^8^	CCS 4	c.2T>A	p.M1?	5 y	+	M	+	III?
This case	−	c.1A>T	p.M1?	3 y	+	M	−	III

months (m), years (y), present (+), absent (−), Severe (S), Moderate (M).

Currently, in the ACMG guidelines, the pathogenicity of start-loss variants is considered very strong evidence of pathogenicity (PVS1), as well as other protein-truncated variants, such as nonsense and frameshift variants^6^. However, it has recently been pointed out that the pathogenicity of start-loss variants may be overestimated and that variants are not necessarily deleterious^12^. Translation initiation can occur at ATG sites located downstream of the native ATG site or even at non-ATG sites^13,14^. Therefore, a start-loss variant may not be deleterious if it has an in-frame ATG site near the original ATG or if the shortening site is not highly conserved among species^12,15^. Interestingly, a previously reported case of CS with a start-loss variant of *ERCC8* [c.2T>A, p.M1?] also showed no characteristic symptoms until 3-year-old and the phenotype was mild, with only slight ataxia and mental language developmental delay at 13-year-old^8,16^. Patients with a start-loss variant of *ERCC8* may exhibit relatively mild symptoms similar to those in the present case. However, the actual translation initiation site of *ERCC8* with the start-loss variant has not yet been identified. It is unclear whether the resulting truncated protein retains partial function; therefore, further studies are required.

CS exhibits a broad clinical spectrum ranging from mild to severe. In particular, CS3 is difficult to diagnose because its characteristic growth failure and physical symptoms are often absent until later in childhood. In the cases of sudden growth failure, CS should be cited as a differential diagnosis, and detailed interviews are necessary for photosensitivity and hearing, as well as comprehensive genetic analysis. It will be possible to diagnose CS at an earlier stage, leading to early detection of complications and genetic counseling.

## HGV database

The relevant data from this Data Report are hosted at the Human Genome Variation Database at: 10.6084/m9.figshare.hgv.3441.
